# The Association between Cardiovascular Risk Factors and Carotid Intima-Media Thickness in 42,726 Adults in UK Biobank: A Cross-Sectional Study

**DOI:** 10.3390/jcdd10090358

**Published:** 2023-08-23

**Authors:** Hanan K. AlGhibiwi, Wedad S. Sarawi, Manal E. Alosaimi, Ahlam M. Alhusaini, Mohammed A. Assiri, Norah K. Algarzae

**Affiliations:** 1 Department of Pharmacology and Toxicology, College of Pharmacy, King Saud University, Riyadh 11149, Saudi Arabia; wsarawi@ksu.edu.sa (W.S.S.); aelhusaini@ksu.edu.sa (A.M.A.); moassiri@ksu.edu.sa (M.A.A.); 2 Department of Basic Health Sciences, College of Medicine, Princess Nourah Bint Abdulrahman University, Riyadh 11671, Saudi Arabia; mealosaimi@pnu.edu.sa; 3 Department of Physiology, College of Medicine, King Saud University, Riyadh 11149, Saudi Arabia; nalgarzae@ksu.edu.sa

**Keywords:** UK Biobank, intima-media thickness, risk, systolic blood pressure, age

## Abstract

Background: Traditional modifiable cardiovascular risk factors, such as high blood pressure, have long been positively correlated with high carotid intima-media thickness (cIMT). However, traditional cardiovascular risk factors made a minor contribution to cIMT variance, meaning that other markers may be regarded as independent markers for increasing cIMT. Aims: To investigate the simple demographic patterns of carotid intima-media thickness (cIMT) in the UK Biobank and to identify which upstream cardiovascular disease (CVD) risk factors are independently associated with cIMT. Methods and Results: A cross-sectional-based study of healthy middle-aged people recruited in the UK between 2006 and 2010 (*n* = 42,726). Results: This study showed that the cardiovascular risk profile generally worsened across the cIMT quantiles from lowest to highest. The lowest cIMT quartile was defined as having a mean cIMT < 588 µm, while the highest cIMT quartile was defined as having a mean cIMT > 748 µm. Specifically, the highest cIMT quantile group had a worse CVD risk factors profile compared to the lowest cIMT quantile group. It was found that, for every one SD increase in age and systolic blood pressure, the mean cIMT increased by 0.357 SD and 0.115 SD, respectively. Conclusion: Systolic blood pressure and age were the strongest independent risk factors for a high cIMT value compared to other risk factors.

## 1. Introduction

Traditional modifiable cardiovascular disease risk factors, including high blood pressure, high blood sugar, high low-density lipoproteins, obesity, and cigarette smoking, have long been positively correlated with high cIMT [1]. More recent studies have illustrated the potential upstream causes of intima thickening. Chiesa et al. (2019) studied cross-sectional associations between a range of CVD risk factors (blood pressure, body composition, lipid, insulin, glucose, inflammatory markers, socioeconomic circumstances and lifestyle behaviours) and cIMT. They found that free fat mass and systolic blood pressure were independently associated with increased cIMT. These findings were derived from a young healthy population, and the sample size was around 5000 participants [2].

Furthermore, an association has been established between cIMT and traditional risk factors, including insulin resistance, dyslipidemia and metabolic syndrome [3,4,5]. However, traditional CVD risk factors made a minor contribution to cIMT variance [6], meaning that other markers may be regarded as independent markers for increasing cIMT. Studies have shown that other biological markers are involved in the inflammation process of intimal hyperplasia, which may be considered independent predictors for increasing cIMT. There is an association between cIMT and other biological inflammatory markers such as c-reactive protein, fibrinogen, apoprotein-B and oxidized LDL [7,8,9,10].

To prevent CVD, it is crucial to understand the upstream causes of vascular disease more clearly. Most existing studies on the determinants of cIMT are based on a limited sample size that focuses on either special clinical populations or a specific age group [11]. Furthermore, most studies have a limited ability to measure confounders as well as the heterogenicity noted between cIMT measurements [12]. This variation in the pre-existing research makes it difficult to draw a robust conclusion.

The UK Biobank is an important resource that provides detailed measurements of cIMT and other cardiovascular risk factors. UK Biobank is a valuable epidemiological resource based on a UK population that includes around 500,000 participants.

In this study, we investigate the upstream determinants for carotid intima-media thickness and the association of cIMT with simple demographic patterns and upstream CVD risk factors, and assess whether these risk factors are independently associated with cIMT.

## 2. Materials and Methods

### 2.1. UK Biobank

UK Biobank is a prospective cohort study recruiting more than 500,000 men and women aged between 40–69 years in the UK between 2006–2010 [13]. Participants attended one of the 22 recruitment centres across the UK, where they performed the baseline physical measurements and gave blood and urine samples for genetic and biomarkers analysis, as well as completing a self-completed touchscreen questionnaire and a computer-assisted personal interview covering a wide range of social and lifestyle information as well as medical conditions.

The imaging study was designed to achieve the target of 100,000 participants. Beginning in May 2014, participants were invited to participate in a follow-up imaging assessment. The imaging scans of vital organs (brain, heart, abdomen, bones, carotid artery, and body composition) and a repeat of the baseline measurements were taken. The study included a cardiac and whole-body MRI, a brain MRI scan, a neck artery ultrasound scan and dual-energy X-ray absorptiometry (DXA). Carotid IMT phenotyping started in 2015 as a pilot phase, during which 2272 individuals were imaged at 18 centres with manual quality control being performed. Subsequently, all imaging centres started to recruit participants and use automated measurements. Recruitment for imaging is ongoing; as of 2019, ultrasound measurements of cIMT had been taken from 502,507 participants [14].

### 2.2. Predictors Measurement (2006–2010)

The date of birth, age and sex were obtained by UK Biobank staff ahead of the assessment visit from local NHS Primary Care Trust Registries. Ethnic group data were collected via patient report during the touchscreen questionnaire during the assessment visit. In this variable, ethnicity was categorised into eight groups (white, black, Asian, mixed, Chinese, other ethnic groups, do not know, and prefer not to answer). This study recorded ethnicity groups as white, south Asian, black, and others. Participants with “Don’t know” and “Prefer not to answer” responses were excluded from our analysis. Blood pressure measurement was conducted at the third station of the assessment centre visit. Systolic and diastolic blood pressure were measured both manually and automatically. The readings for blood pressure were taken twice during the imaging visit at the assessment centre by qualified nurses. An Omron 705 IT electronic blood pressure monitor was used to measure blood pressure (OMRON Healthcare Europe B.V. Kruisweg 577 2132 NA Hoofddorp, OMRON Healthcare, Netherlands, Europe). There was only an initial and first repeat assessment visit for blood biochemistry and no assessment for blood components at the imaging visit that measured cIMT, and this is one of the study’s limitations.

We defined blood pressure preferentially using automated or manual measurements where automated measurements were unavailable. Body size measures, including height, weight, waist, and hip circumference, were taken by UK Biobank staff during the assessments at the assessment centre. Weight was measured using the Tanita BC-418 AM body composition analyser and height using the Seca 202 height measure. UK Biobank staff asked participants to remove heavy clothes and shoes and stand on the footpads of the body composition analyser. Body mass index was calculated by dividing the weight by the square of the height. Waist and hip circumferences were measured using a Wessex non-stretchable sprung tape measure.

UK Biobank measured selected biochemical markers in biological samples, including serum, urine and saliva. These samples were collected at baseline (2006–2010) for all 500,000 participants. Around 34 biomarkers were selected to be analysed because they represented disease risk factors and were considered diagnostic biomarkers. Blood samples were also taken at the imaging visit, but these have not been analysed to date. For this reason, the present study uses biochemistry measurements for each participant from 2006–2010, which is at the study baseline assessment and was several years before the imaging visit. For the present study, participants were identified as having diabetes if they reported yes to the ‘diabetes diagnosed by doctor’ variable. During the imaging visit (2014–2018), participants completed a touchscreen questionnaire asking “has a doctor ever told that you have diabetes?”. This was supplemented by information from the nurse interview of self-reported type 1 or type 2 diabetes or the use of insulin drugs. Participants were identified as having hypertension if they reported taking blood pressure medications. During the imaging visit (2014–2018), participants were asked (via touchscreen questionnaire) “Do you regularly take any of the following medications?” The choices included (cholesterol-lowering medications, blood pressure medication, and insulin.

During the imaging visit (2014–2018), participants completed a touchscreen questionnaire. In this study, we grouped the participants into ‘never smokers’, ‘former smokers’ and ‘current smokers’ based on their responses to a touchscreen questionnaire on *smoking habits.* For the question ‘Do you smoke now?’, those who answered ‘Yes’ were categorised as ‘current smokers’. Participants who answered ‘No’ were further asked whether they had previously smoked. Participants who had previously smoked were categorised as ‘former smokers’, and those who were neither previously smoked nor had a current smoker were categorised as ‘never smokers.’ [15].

Participants indicating that they took regular cholesterol-lowering medications were asked by an interviewer for specific details about the class of medication. The Townsend deprivation index is a measure of material deprivation within a population that was first introduced by sociologist Peter Townsend in 1987. It is measured by including four variables: unemployment, non-car ownership, non-home ownership, and household overcrowding, for the population of each area, and a score is given accordingly. The higher the Townsend index score, the greater the degree of deprivation in this area. Townsend deprivation index was measured for each participant joining the UK Biobank. The score was calculated using the preceding national census output areas and their postal code.

Baseline CVD was defined as cardiovascular disease (coronary heart disease, angina, stroke or transient ischaemic attack) self-reported by touchscreen or at the nurse-led participant interview. This was used as part of the exclusion criteria for the present cross-sectional study.

### 2.3. Outcomes Measurement (2014–2018)

Carotid intima-media thickness was measured using a CardioHealth Station (Panasonic Biomedical Sales Europe BV, Leicestershire, UK). The participants were asked to lie down with their head at a 45° angle relative to the horizontal plane and supported by a triangular pillow. The right side was scanned first, followed by the left side. A 2D scan was first performed on the transverse plane (short axis) from below the carotid bifurcation to below the jaw. A 2D scan was then performed for the longitudinal plane (long axis). The carotid intima-media thickness was measured at two predefined angles for each carotid, giving a total of four cIMT readings (right 150°, right 120°, left 210°, and left 240°) at the long axis. The distal common carotid was scanned, and the flow divider between the external and internal carotid artery was located. The far wall of the common carotid was tracked within the box, and after several cardiac cycles, the image auto-freezes in end-diastole. A mean, maximum and minimum of the cIMT tracking was measured and recorded for each acquisition angle for each carotid. The average mean common cIMT was calculated for each individual using the maximum set of carotid angles, near or far wall measurements and left or right side measurements [16].

The measurement of IMT at one site may differ from measurements taken from another IMT site because of the focal nature of the atherosclerosis development in the artery [17]. Therefore, measurements of carotid intima-media thickness from a single site may decrease the sensitivity of detection of the atherosclerosis change in the artery. Some studies evaluate cIMT from a single angle [18], while others provide images from multiple angles. Imaging cIMT from multiple angles is considered a better way of evaluating the three dimensions of the carotid artery [19]. This study examined the mean cIMT at each angle—left, right and combined angles.

**Figure 1:**
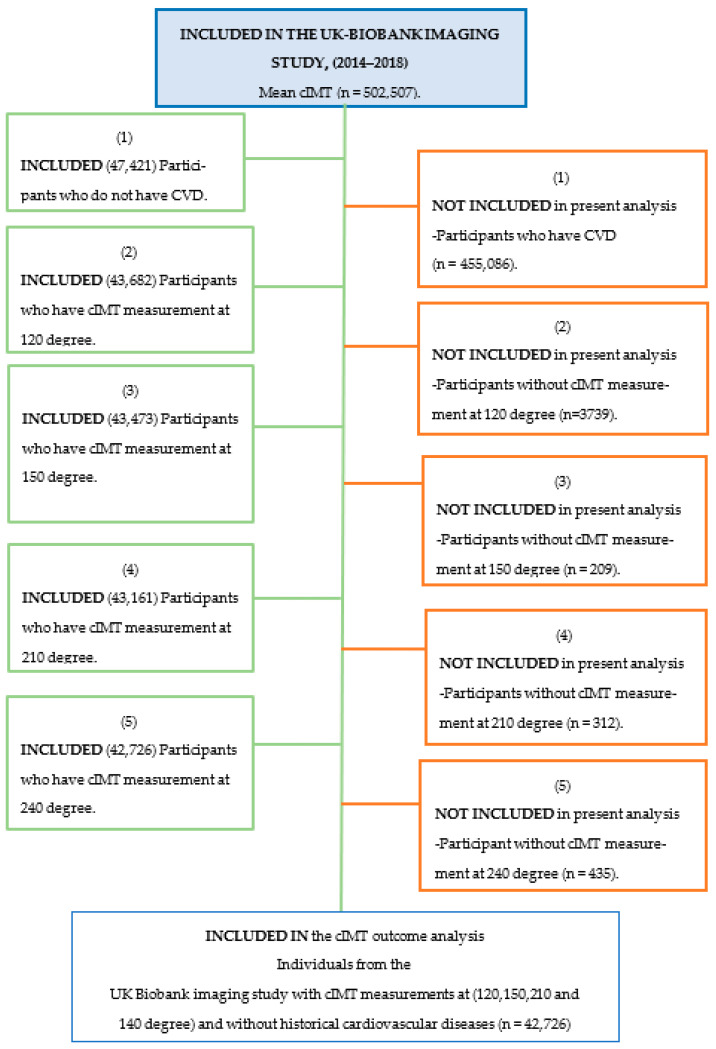
Diagram for inclusion of participants has measurements of cIMT at imaging study.

The analysis included all participants with valid cIMT measurements at all angles and excluded participants with one or more mean cIMT measurements missing. Participants with pre-existing cardiovascular disease were excluded. The boundaries of IMT quartiles were cIMT of <0.58 mm, cIMT (0.59–0.66), cIMT (0.66–0.75), and cIMT > 0.75, (Figure 1).

### 2.4. Statistical Analyses

The distribution of baseline characteristics was investigated across cIMT quartiles ((150.5–594 µm), (594.25–665 µm), (665.25–756.75 µm), and (757–2126.25 µm)), and displayed as: mean (standard deviation) if normal; median (interquartile range (IQR)) if skewed; or number (percentage) if categorical. Comparisons of means between groups were performed using one-way analysis of variance, Kruskal-Wallis, and χ2, respectively.

The Pearson correlation coefficient was used to correlate cardiovascular risk factors and cIMT. Variables with significant correlations with outcomes were subsequently included in a simple linear regression analysis. If associations were seen, they were included in a multiple linear regression analysis to predict the outcomes. *p*-values of 0.05 were used as the cut-off for statistical significance. All analyses were conducted using SPSS 24.

Univariate and multivariate analyses were used to evaluate and adjust the effect of cIMT determinants on the outcome. As the dependent variable was continuous, linear regression was used. The assumptions of constant variance of the residuals, normally distributed residuals, and the linearity of the association were examined by visual inspection of the diagnostic plots. The independent variables were a mix of binary categorical variables with more than two groups and continuous variables. Each predictor was entered into a simple linear regression analysis; variables that were associated with the outcome significantly (*p* < 0.05) in the univariate analysis were subsequently entered into the multiple linear regression. If two variables were significantly correlated, one (the weakest) was excluded from the multiple model as these variables had an association with the outcome due to their relationships with each other. In building multivariable models, first, all categorical and continuous variables significantly associated with cIMT in univariable analysis were subjected to multicollinearity diagnosis, and the variable that had variance inflation factors (VIF) of more than 2 was excluded from the model. No correction was made for multiple testing in regression analysis (i.e., significant test *p* < 0.05).

### 2.5. Patient Involvement

No Patient and/or public were involved in the design and implementation of the study, nor were they involved in conducting the study, interpretation, or report of results.

## 3. Results

### 3.1. Baseline Characteristics

Table 1 gives the baseline characteristics of 42,726 participants from the UK Biobank. The 25th, 50th, 75th percentiles of the summary cIMT measure were 594, 665, and 757, respectively; these cut-off points were used to categorize the cIMT quantiles. The cardiovascular risk profile worsened across the cIMT quantiles from lowest to highest. Specifically, the highest cIMT quantiles group had a worse CVD risk factors profile compared to the lowest cIMT quantile, including age (9 years older), systolic blood pressure (16 mmHg higher), diastolic blood pressure (0.9 mmHg higher), glucose (0.09 mmol/L higher), HbA1c (1.78 mmol/mol higher), hip circumference (1 cm higher), waist circumference (5 cm higher) and BMI (1 kg/m^2^ higher). In addition, subjects in the highest cIMT quantile groups had a poorer lipid profile compared to the lowest cIMT quantile group. Regarding liver enzymes, subjects in the highest cIMT quantile group had higher alanine aminotransferase and aspartate aminotransferase values than the lowest cIMT quantile group. Additionally, they had a higher C-reactive protein value and vitamin D value. The prevalence of diabetes and hypertension in this study population was significantly higher in the highest cIMT quantile group compared to the lowest cIMT quantile group.

Furthermore, there was a significantly higher percentage of subjects who had received statin therapy in the highest cIMT quantile group compared to the lowest cIMT quantile group. In comparison with the lower cIMT quantile group, there was a higher proportion of subjects who were former smokers in the highest quantile group (39.8%) compared to the lowest quantile group (28.2%). The majority of participants were white in each of the cIMT quantiles. In addition, there was a higher percentage of males (75.2%) compared to females (24.8%) in the highest quantile group.

### 3.2. The Association between cIMT and Cardiovascular Risk Factors

#### 3.2.1. Correlations

Bivariate Pearson correlation was carried out among all continuous variables. Mean cIMT showed a significant positive linear association with HbA1c, diastolic blood pressure, cholesterol, triglyceride, LDL and liver enzymes, c-reactive protein, apolipoprotein B vitamin D, and anthropometric measurements. However, a negative linear relationship existed between cIMT and other CVD risk factors, including HDL, apoprotein A and Townsend deprivation index. Age and systolic blood pressure showed the strongest correlation with mean cIMT (r = 0.413, r = 0.307) compared to other CVD risk factors (Table 2).

#### 3.2.2. CIMT, Single Factor Analysis in the Whole Cohort

To identify the predictors of cIMT in all subjects, univariable regression analysis was carried out for all variables that met statistical significance in bivariate Pearson correlation.

When ranked by standardised beta coefficients, age was the most important univariable determinant for cIMT (B = +0.413). Then, systolic blood pressure (B = +0.307). After that, male (B = +0.18) and waist circumference (B = +0.164). The results are shown in Table 3 for univariable analysis for CVD risk factors and cIMT.

#### 3.2.3. Multiple Regression Model of cIMT with Cardiovascular Risk Factors

This section identified the covariables associated with cIMT in adjusted models (multivariate), as shown in Table 3. The stepwise model ended with these factors: age, gender, systolic blood pressure, diabetes, hip circumference, vitamin D, apolipoprotein-A, apolipoprotein-B, hypertension and smoking. Following this, waist circumference was dropped due to collinearity.

After performing a stepwise multiple regression model, the risk factors identified as the strongest predictive factor for cIMT were: age (for every 1 SD increase in age, the mean cIMT increases by (0.357 μm) SD), systolic blood pressure (for every 1 SD increase in systolic blood pressure, the mean cIMT increases by (0.115 μm) SD) and sex (mean cIMT increases by (0.124 μm) SD in male subjects compared with females). Overall, this model, including age, gender, systolic blood pressure, diabetes, hip circumference, vitamin D, apolipoprotein-A, apolipoprotein-B, hypertension, and smoking, explained 22% of the variance in cIMT in the UK Biobank population.

## 4. Discussion

### 4.1. Principal Findings

This cross-sectional study found that the strongest determinants for cIMT were age and systolic blood pressure, which, in combination with the other risk factors shown in Table 3, explained 21.6% of cIMT variation.

This study showed that cIMT values increased linearly with age in all study participants. The results of this study are also in line with data from a large US-based study: Atherosclerosis Risk in Communities. The cross-sectional association of mean IMT as a function of age in 4952 individuals was estimated, and a data study showed that cIMT increases with age in all carotid segments [20]. In addition, a study by Maria Łoboz-Rudnicka [21] found that mean cIMT increased with age in both men and women.

The second strongest determinant of cIMT in the current study was systolic blood pressure. The study showed that there was a linear association between high systolic blood pressure and increased cIMT values in all study participants in single and multiple analyses. Diastolic blood pressure was only weakly associated with cIMT in univariable models and was not included in the stepwise model. In line with the findings of this study, the STANISALS cohort study, which is a single, cross-sectional, population-based study based on the Nancy region of France, involving 696 adult participants, found the risk of having cIMT > 900 µm linearly increased with increased systolic blood pressure, and there was a weaker association between diastolic blood pressure and cIMT [22].

Another population-based cross-sectional study among low-income adults in rural China, involving 2643 normal participants, of whom 549 participants were from an impaired fasting glucose group and 533 were diabetic, reported a significant association between higher cIMT values and higher systolic blood pressure: diastolic blood pressure was found to be a protective factor from increasing cIMT in multiple analyses [23]. Likewise, another study showed a significant positive association between systolic blood pressure and cIMT, but this association was not reported with diastolic blood pressure [24]. These findings have also been documented in other studies [25], and suggest that SBP may induce a higher pressure overload, an thus, more hyperplasia and hypertrophy than DBP. Moreover, some authors debated that SBP may be more important than DBP as a risk factor for cardiovascular diseases and atherosclerosis [25,26]. Several studies have discussed the role of hypertension in structural remodelling characterised by carotidintima-media thickness. Roman et al. examined the presence of structural changes in carotid artery in 43 hypertensive patients and 43 control subjects, and they found that high systolic blood pressure was a stronger predictor for increased cIMT [27]. Another study detects a significant and independent positive association between carotid IMT, systolic blood pressure and age, as well as an association between cIMT and cardiac remodelling. This study is a sub-study from a large multicentre cross-sectional study, the ISMIR (Ispessimento Medio-Intimale e Rischio Cardiovascolare), involving 198 asymptomatic, never treated, essential hypertensives and 67 healthy subjects [28].

Our data substantiate the theory that targeted organ injury is primarily due to an increase in hemodynamic burden [29]. Artery wall thickening and cardiac remodelling depend on hemodynamic change [30], and several mechanisms could explain the association between them. Elevation of systolic blood pressure usually accompanies reduced arterial compliance, leading to increased heart afterload, which may cause cardiac hypertrophy [31]. As shown in the results of this study, systolic blood pressure was significantly higher (146 ± 19) for individuals in the highest cIMT quartile. In contrast to our findings, The Kuopio Ischaemic Heart Disease Risk Factor Study found that hypertension was not associated with increased IMT in 720 men [32].

Moreover, subsequent data from the same author did detect a significant association between systolic blood pressure and increased cIMT in 1224 men [33]. It is likely that selection criteria (based on age or blood pressure restrictions) have some impact on the strength of observed associations. UK Biobank has the advantage of comprising a wide range of ages and blood pressures among broadly healthy participants.

This study reported that traditional risk factors, such as age, systolic blood pressure, BMI, LDL, and waist circumference, as well as less traditional factors, including apolipoprotein A and apolipoprotein B, explained 21.6% of the cIMT variance. This means that other factors that were not included in this study may have contributed to cIMT variance. Studies showed that the lifestyle factors such as alcohol consumption, dietary habits and physical activity, as well as job stress and cognitive performance, are associated with cIMT variance [34,35,36]. The early screening of cIMT and more aggressive management are warranted for patients with hypertension, especially if they are elderly, have central obesity and have high levels of glucose and lipid.

However, the value added by cIMT measurements above risk factors included in the Framingham Risk Score is conflicting [37]. The ESH/ESC were more cautious in their recommendation for cIMT measurement of patients with hypertension in guidelines released in 2013 [38] than their release in 2007 [39]. In addition, the result from IMPROVE (Immediate Management of the Patient with Ruptured Aneurysm: Open Versus Endovascular Repair) cohort study showed that insignificant c-statistic predicted stroke or myocardial infarction after adding cIMT to the Framingham Risk Score [40].

### 4.2. Strengths and Limitations

The major strengths of this study are its large sample size; it was a well-designed cohort study that included several markers, which enabled the researchers to study the association between a wide range of CVD risk factors and outcomes. In addition, all measurements were taken in a standardised and unified way in all participants of UK Biobank. However, some limitations to the findings are acknowledged. There was only an initial and first repeat assessment visit for blood biochemistry and blood pressure; no blood was taken at the imaging visit. Therefore, on average, 4 years passed between these measurements. Changes to blood biochemistry (such as lipids) and blood pressure in the intervening period may have contributed to the small percentage that classical factors accounted for imaging markers (cIMT) variation. However, as the effects of classical risk factors would be cumulative over time, and in general, they would be stable or worsen with age, it is reasonable to have hypothesised that these factors would have contributed to vascular markers of cardiovascular disease measured, on average, 4 years later. This Biobank study included participants aged between 40–70 years, and the findings may not be generalizable to other populations. As the study is cross-sectional, causality cannot be inferred. The selection of determinants to be investigated might be limited with respect to other socioeconomic or sociocultural characteristics. However, the aim was to include biological-related risk factors for atherosclerosis, which were available in this study. The work requires external validation and will increase in significance as more participants undergo imaging visits.

## 5. Conclusions

Our study has shown the upstream determinants for cIMT in a large UK Biobank study. Understanding these factors might help clinicians in the primordial prevention of the development and progression of subclinical atherosclerosis, which is considered an early indicator of atherosclerosis.

## Figures and Tables

**Table 1:** Characteristics of the study population stratified according to cIMT quantiles.

Continuous Variables		Mean cIMT (150.5–594) µm Number (mean ± SD)	Mean cIMT (594.25–665) µm Number (mean ± SD)	Mean cIMT (665.25–756.75) µm Number (mean ± SD)	Mean cIMT (757–2126.25) µm Number (mean ± SD)
**Non-invasive measurement of atherosclerosis**					
**Mean cIMT**		(*n* = 10,701), 546 ± 35.1	(*n* = 10,663), 629 ± 20	(*n* = 10,692), 708 ± 26	(*n* = 10,670), 856 ± 91
Pulse wave arterial stiffness index *		(*n* = 9058), 9.03 (7.26, 10.86)	(*n* = 8982), 9.35 (7.38, 11.20)	(*n* = 8910), 9.61 (7.56, 11.54)	(*n* = 8913), 9.83 (7.74, 11.86)
**Demographic data**					
**Age (year)**		(*n* = 10,701), 59.3 ± 7	(*n* = 10,663), 62.7 ± 7.1	(*n* = 10,692), 65.5 ± 7	(*n* = 10,670), 67.9 ± 6.6
**Townsend deprivation index**		(*n* = 10,686), −1.77 ± 2.78	(*n* = 10,650), −1.86 ± 2.72	(*n* = 10,685), −1.97 ± 2.70	(*n* = 10,663), −1.98 ± 2.69
**Anthropometric measurements**					
**Waist circumference (cm)**		(*n* = 10,417), 85.5 ± 12.25	(*n* = 10,368), 86.8 ± 12.12	(*n* = 10,358), 88.3 ± 12.55	(*n* = 10,299), 91 ± 12.19
**Hip circumference (cm)**		(*n* = 10,418), 99.89 ± 8.75	(*n* = 10,368), 100.47 ± 8.62	(*n* = 10,358), 100.88 ± 8.68	(*n* = 10,299), 101.8 ± 8.36
**BMI (kg/m^2^)**		(*n* = 10,385), 25.9 ± 4.29	(*n* = 10,344), 26.3 ± 4.25	(*n* = 10,324), 26.5 ± 4.32	(*n* = 10,265), 26.9 ± 4.25
**Blood pressure measurements**					
**Systolic blood pressure (mmHg)**		(*n* = 9522), 130 ± 16.4	(*n* = 9466), 136 ± 17.4	(*n* = 9351), 140 ± 18	(*n* = 9325), 146 ± 18.9
**Diastolic blood pressure**		(*n* = 9522), 78 ± 9.7	(*n* = 9466), 78.9 ± 10	(*n* = 9351), 78.9 ± 10	(*n* = 9325), 78.9 ± 10
**Diabetic measurements**					
Glucose *(mmol/mol)		(*n* = 9069), 4.83 (4.53, 5.16)	(*n* = 9128), 4.87 (4.55, 5.21)	(*n* = 9096), 4.89 (4.58, 5.23)	(*n* = 9134), 4.92 (4.60, 5.28)
**HbA1c (mmol/mol)**		(*n* = 9931), 34 ± 4.41	(*n* = 9877), 34.77 ± 4.87	(*n* = 9962), 35.22 ± 5.20	(*n* = 9906), 35.78 ± 5.67
**Enzyme measurements**					
Alanine aminotransferase * (U/L)		(*n* = 9987), 18.31 (14, 25.22)	(*n* = 9979), 18.9 (14.67, 25.86)	(*n* = 10,004), 19.98 (15.36, 26.83)	(*n* = 9988), 21 (16.43, 28.09)
Aspartate aminotransferase * (U/L)		(*n* = 9938), 23.2 (20.10, 27.50)	(*n* = 9943), 23.80 (20.5, 28)	(*n* = 9966), 24.40 (21, 28.50)	(*n* = 9957), 25 (21.70, 29.20)
**Lipid measurements**					
**Total** **cholesterol (mmol/L)**		(*n* = 9988), 5.6 ± 1.04	(*n* = 9983), 5.7 ± 1.07	(*n* = 10,006), 5.78 ± 1.07	(*n* = 9988), 5.83 ± 1.12
**HDL-cholesterol (mmol/L)**		(*n* = 9080), 1.50 ± 0.37	(*n* = 9136), 1.51 ± 0.38	(*n* = 9105), 1.49 ± 0.38	(*n* = 9143), 1.42 ± 0.36
**LDL-cholesterol (mmol/L)**		(*n* = 9969), 3.47 ± 0.80	(*n* = 9962), 3.56 ± 0.82	(*n* = 9980), 3.61 ± 0.82	(*n* = 9964), 3.73 ± 0.85
Triglyceride *		(*n* ≈ 9980), 1.26 (0.91, 1.86)	(*n* ≈ 9975), 1.34 (0.95, 1.92)	(*n* ≈ 10,001), 1.42 (1.02, 2.04)	(*n* = 9979), 1.53 (1.10, 2.19)
**Apolipoprotein-A(g/L)**		(*n* = 9024), 1.56 ± 0.26	(*n* = 9086), 1.56 ± 0.27	(*n* = 9055), 1.56 ± 0.27	(*n* = 9102), 1.51 ± 0.25
**Apolipoprotein-B (g/L)**		(*n* = 9933), 1 ± 0.22	(*n* = 9927), 1.02 ± 0.23	(*n* = 9956), 1.04 ± 0.23	(*n* = 9959), 1.07 ± 0.23
**Inflammatory markers**					
C-reactive protein (mg/L) *		(*n* = 9969), 0.95 (0.48, 1.94)	(*n* = 9960), 1.02 (0.53, 2.06)	(*n* = 9983), 1.11 (0.57, 2.20)	(*n* = 9968), 2.18 (1.2, 3.63)
**Vitamins**					
Vitamin D * (nmol/L)		(*n* = 9680), 46.20 (32.30, 62.10)	(*n* = 9611), 48.20 (33.80, 63.20)	(*n* = 9589), 48.80 (34.60, 63.50)	(*n* = 9519), 48.90 (34.40, 64.10)
**Categorical variables**		(number,%)	(number,%)	(number,%)	(number,%)
**Diabetes**	No	10,327 (96.5%)	10,198 (95.6%)	10,089 (94.4%)	9953 (93.3%)
	Yes	374 (3.5%)	465 (4.4%)	603 (5.6%)	717 (6.7%)
**Hypertension**	No	6130 (57.3%)	4839 (45.4%)	3946 (36.9%)	2787 (26.1%)
	Yes	4571 (42.7%)	5824 (54.6%)	6746 (63.1%)	7883 (73.9%)
**Smoking**	Never	7237 (68.1%)	6802 (64.4%)	6519 (61.6%)	5955 (56.6%)
	Former	3002 (28.2%)	3386 (32.1%)	3728 (35.2%)	4183 (39.8%)
	Current	388 (3.7%)	376 (3.6%)	336 (3.2%)	381 (3.6%)
**Statin**	No	9122 (85.2%)	8498 (79.7%)	8003 (74.9%)	7284 (68.3%)
	Yes	1579 (14.8%)	2165 (20.3%)	2689 (25.1%)	3386 (31.7%)
**Ethnicity**	White	10,280 (96.3%)	10,316 (97%)	10,395 (97.5%)	10,350 (97.3%)
	Black	52 (0.5%)	67 (0.6%)	68 (0.6%)	94 (0.9%)
	South Asian	134 (1.3%)	99 (0.9%)	78 (0.7%)	72 (0.7%)
	Other	211 (2%)	156 (1.5%)	124 (1.2%)	122 (1.1%)
**Sex**	Female	4305 (61.7%)	4715 (58%)	3658 (43.1%)	108 (24.8%)
	Male	2673 (38.3%)	3414 (42%)	4831 (56.9%)	328 (75.2%)

Data are expressed as number (mean ± SD) for normal distribution, * non-normal distribution data are expressed as number, median (Q1, Q3). Categorical data are expressed as account (percentage). All variables globally significantly different between groups at *p* < 0.001.

**Table 2:** Heatmap representation of the Pearson correlation coefficients of mean cIMT with clinical characteristics in the whole cohort.

Continuous Variables	Mean cIMT		
	Pearson Correlation (r)	*p*-Value	
Age (year)	0.4129	<0.001	1
Systolic blood pressure (mmHg)	0.3074	<0.001	
Waist circumference (cm)	0.1648	<0.001	
Log-Triglycerides(mmol/L)	0.129	<0.001	
Apolipoprotein-B (g/L)	0.1248	<0.001	
HbA1c (mmol/mol)	0.1221	<0.001	
LDL cholesterol (mmol/L)	0.1041	<0.001	
Log-Alanine aminotransferase (U/L)	0.1035	<0.001	
Log-Aspartate aminotransferase (U/L)	0.0974	<0.001	
BMI (kg/m^2^)	0.0847	<0.001	
Log-C-reactive protein(mg/L)	0.0801	<0.001	
Total cholesterol (mmol/L)	0.0766	<0.001	
log-Glucose (mmol/L)	0.0663	<0.001	
Hip circumference (cm)	0.059	<0.001	
Pulse wave velocity at baseline (m/s)	0.055	<0.001	
Log Vitamin D (nmol/L)	0.0414	<0.001	
Diastolic blood pressure (mmHg)	0.0264	<0.001	
Townsend deprivation index at recruitment	−0.0309	<0.001	
Apolipoprotein-A (g/L)	−0.0675	<0.001	
HDL cholesterol (mmol/L)	−0.0925	<0.001	−1

r: Pearson correlation coefficient. BMI: body mass index, HbA1c: glycated haemoglobin, LDL: low density lipoprotein, HDL: high density lipoprotein.

**Table 3:** Factors associated with carotid intimal media thickness (cIMT) differences among all study participants (*n* = 42,726).

Variables	Simple Linear Regression			Stepwise Regression Analysis	
		B (95% CI)	(b)	B (95% CI)	(b)
**Age (year)**		6.773, (6.631, 6.915)	0.413	5.87, (5.66, 6.09)	0.357
**Anthropometric measurements**					
**Waist circumference(cm)**		1.648, (1.553, 1.743)	0.164		
**Hip circumference(cm)**		0.854, (0.715, 0.993)	0.058	0.744, (0.544, 0.945)	0.049
**BMI (kg/m^2^)**		2.465, (2.185, 2.745)	0.084		
**Blood pressure measurements**					
**Systolic blood pressure (mmHg)**		2.06, (1.997, 2.126)	0.307	0.77, (0.678, 0.862)	0.115
**Diastolic blood pressure(mmHg)**		0.327, (0.201, 0.452)	0.026		
**Diabetic measurements**					
Log-Glucose * (mmol/L)		55.21, (46.67, 63.74)	0.066		
**HbA1c (mmol/mol)**		2.999, (2.75, 3.23)	0.122		
**Enzymes measurements**					
Log-Alanine aminotransferase * (U/L)		28.69, (25.98, 31.39)	0.103		
Log-Aspartate aminotransferase * (U/L)		46.14, (41.51, 50.77)	0.097		
**Lipid measurements**					
**Total cholesterol (mmol/L)**		8.86, (7.73, 9.99)	0.076		
**HDL cholesterol (mmol/L)**		−30.70, (−34.10, −27.31)	−0.092		
**LDL cholesterol (mmol/L)**		15.73, (14.25, 17.20)	0.104		
**Apolipoprotein-A (g/L)**		−31.94, (−36.79, −27.08)	−0.067	−25.224 (−31.970, −18.47)	−0.053
**Apolipoprotein-B (g/L)**		68.240, (62.906, 73.574)	0.124	32.99, (25.27, 40.71)	0.055
**Log-Triglycerides (mmol/L)**		70, (69, 72)	0.129		
**Other markers**					
**Log-C-reactive-** Protein (mg/L) *		9.847, (8.644, 11.051)	0.080		
Log-Vitamin D * (nmol/L)		−1.420, (−1.855, −0.984)	−0.031	0.169, (0.093, 0.245)	0.029
**Categorical variables**					
**Diabetes**	Reference	683, (681, 684.37)	0.0634	10.97, (3.50, 18.45)	0.019
	Yes	36.232, (30.82, 41.63)			
**Hypertension**	Reference	650, (648.37, 651.95)	0.234	8.76, (5.11, 12.40)	0.034
	Yes	59.46, (57.12, 61.80)			
**Smoking**	Reference	675.42, (673.93, 676.91)	0.099	10.96, (8.33, 13.59)	0.0418
	Former	26.13, (23.61, 28.66)	0.0151		
	Current	10.28, (3.78, 28.66)		21.49, (14.77, 28.20)	0.0317
**Ethnicity**	Reference	685.43, (684.22, 684.22)	0.0162		
	Black	25.06, (10.40, 39.72)			
	South Asian	−28.35, (−40.93, −15.78)	−0.0214		
	Other	−27.31, (−37.28, −17.35)	−0.026		
**Statin**	Reference	674, (673.38, 676.05)	0.150		
	Yes	44.69, (41.90, 47.48)			
**Gender**	Reference	663, (661.76, 664.98)	0.18	25.50, (21.04, 27.04)	0.124
	Male	45.52, (43.18, 47.85)			
				R^2^%	21.6%

95% CI: 95% confidence interval. B: beta coefficients, per unit increase of predictor. Standardized (b) coefficient: mean change in cIMT (µm) per 1 SD increase in predictor variable. Stepwise (Criteria: Probability of-F-to enter ≤ 0.050, Probability of-F-to-remove ≥ 0.100).

## Data Availability

Researchers can apply to use the UK Biobank resource and access the data used. No additional data are available.
